# Quantification of mitochondrial DNA damage and copy number in circulating blood of patients with systemic sclerosis by a qPCR-based assay^☆^^☆☆^

**DOI:** 10.1016/j.abd.2019.11.003

**Published:** 2020-03-20

**Authors:** Shafieh Movassaghi, Sara Jafari, Kowsar Falahati, Mitra Ataei, Mohammad Hossein Sanati, Zohreh Jadali

**Affiliations:** a Department of Rheumatology, Imam Khomeini Hospital, Tehran University of Medical Sciences, Tehran, Iran; b Clinical Genetics Department, National Institute of Genetic Engineering and Biotechnology, Tehran, Iran; c School of Public Health, Tehran University of Medical Sciences, Tehran, Iran

**Keywords:** Autoimmunity, DNA, Mitochondrial, Oxidative stress, Real-time polymerase chain reaction, Scleroderma, Systemic

## Abstract

**Background:**

Although not fully understood, oxidative stress has been implicated in the pathogenesis of different autoimmune diseases such as systemic sclerosis. Accumulating evidence indicates that oxidative stress can induce mitochondrial DNA (mtDNA) damage and variations in mtDNA copy number (mtDNAcn).

**Objective:**

The aim of this study was to explore mtDNAcn and oxidative DNA damage byproducts in peripheral blood of patients with systemic sclerosis and healthy controls.

**Methods:**

Forty six patients with systemic sclerosis and forty nine healthy subjects were studied. Quantitative real-time PCR used to measure the relative mtDNAcn and the oxidative damage (oxidized purines) of each sample.

**Results:**

The mean mtDNAcn was lower in patients with systemic sclerosis than in healthy controls whereas the degree of mtDNA damage was significantly higher in cases as compared to controls. Moreover, there was a negative correlation between mtDNAcn and oxidative DNA damage.

**Study limitations:**

The lack of simultaneous analysis and quantification of DNA oxidative damage markers in serum or urine of patients with systemic sclerosis and healthy controls.

**Conclusion:**

These data suggest that alteration in mtDNAcn and increased oxidative DNA damage may be involved in the pathogenesis of systemic sclerosis.

## Introduction

Systemic Sclerosis (SSc) is a rare chronic disease of connective tissue characterized by a vasculopathy and fibrosis in the skin and various internal organs.^1^, ^2^ The exact cause of disease is unknown, but its occurrence can be influenced by a complicated interplay of genetic, epigenetic and environmental factors.^3^ Recently, great attention has been devoted to the role of oxidative stress in the pathogenesis of SSc. It is thought to be involved in the pathophysiological processes of increased synthesis and accumulation of extracellular matrix, abnormal immune activation and vascular endothelial damage, the three outstanding features of disease.^4^, ^5^

Increased oxidative stress reflects an imbalance between pro-oxidant/antioxidant homeostasis and leads to increased levels of toxic Reactive Oxygen Species (ROS). There are two main pathways of ROS generation, the endogenous and exogenous pathways. Mitochondria are the major endogenous source of ROS. They have their own genome [mitochondrial DNA (mtDNA)] and are indispensable organelles for normal cellular physiology. mtDNA is multi-copy, and its abundance is regulated in a tissue specific manner. mtDNAcn fluctuates in response to the physiological environment surrounding the cell, but remains relatively stable within cells under physiological conditions.^6^ In pathological circumstances, the number of mtDNAcn can vary depending on different factors, including altered cellular redox balance. mtDNA is also prone to oxidative stress damage because of its high exposure to the ROS that are produced by mitochondria.^7^

Guanine oxidation products are important indicators of oxidative DNA damage. These chemical markers can be removed by Fpg (formamidopyrimidine [fapy]-DNA glycosylase), which is a base excision repair enzyme and releases purine lesions from both nuclear and mtDNA.^7^, ^8^ Fpg cleavage of oxidatively damaged DNA can reduce amplification of enzyme-digested target DNA by Taq DNA polymerase.^9^ Therefore, this method has been widely used as a measure of oxidative damage to DNA. The aims of present study were to determine whether mtDNAcn variation and its oxidative damage index – mtDNA(ΔCT); can be detected in patients with SSc.

## Methods

The study has received approval from the ethics committee of University of Medical Sciences, and was performed in accordance to the ethics principles in the Declaration of Helsinki. All participants voluntarily agreed to participate in the study and gave fully informed consent.

### Study population

Forty six patients with SSc and 49 age and sex matched healthy individuals with no history of scleroderma or other chronic and autoimmune diseases were included in the study. The patients with SSc were diagnosed according to the American College of Rheumatology (ACR)/European League Against Rheumatism (EULAR) 2013 criteria.^10^, ^11^ Among the enrolled patients, 6 (13.04%) had limited form of SSc and 40 (86.96%) had diffuse systemic sclerosis. 12 (26.09%) had interstitial lung disease (ILD), 3 (6.52%) had Pulmonary Arterial Hypertension (PAH) and 3 (6.52%) had both ILD and PAH. The mean age of the patients with SSc (6 men, 40 women) and the controls (5 men, 44 women) was 46.09 and 41.90 years, respectively. The mean duration of disease was 7.61 ± 6.13 years with a range of 1–40 years. Thirty eight patients with diffuse systemic sclerosis and 6 patients with limited cutaneous SSc had received steroid and/or immunosuppressant treatments before sampling. Drug dose and duration differs depending on the severity of the lesions and the affected organs. None of the patients had inflammatory/autoimmune disorders other than SSc and all of them were in an inactive state at the time of visit. Table 1 shows demographic and clinical characteristics in SSc subjects and controls.

**Table 1:** Demographic and clinical features of patients with systemic sclerosis and healthy controls.

Features	Patients		Controls
*Number*	46		49
*Age (years)*	46.09 ± 12.70		41.90 ± 12.23
*Duration of disease (years)*	7.61 ± 6.13		
*Gender (female/male)*	40/6		44/5
*IcSSc/dcSSc, n (%)*	6 (13.04%)/40 (86.96%)		0

*Organ involvement*			
Skin	30		0
Lung (ILD)	17		0
Heart (PAH)	10		0
Renal (history of renal crsis)	1		0
Gastrointestinal	20		0
Joint	5		0

*Treatment*			
Type of disease	Drug name	Number of patients	
Limited cutaneous systematic sclerosis	Methotrexate	4	
	Azatioprin	1	
	Mycophenolate	1	
Diffuse systemic sclerosis	Methotrexate	6	
	Azatioprin	10	
	Mycophenolate	14	
	Prednisolone	1	
	Azatioprin + prednisolone	7	
	None	2	

### DNA extraction

Blood samples were collected into 5 mL tubes containing ethylene diamino tetraacetic acid (EDTA). DNA extraction from whole blood was performed using DNA isolation kit (GeneAll, Seoul, Korea), according to the manufacturer's instructions.

The concentration and purification of the extracted DNA were measured by using agarose gel electrophoresis (1.5%) and spectrophotometry with NanoDrop 2000c (Thermo Fisher Scientific, USA). All DNA samples were subsequently stored at −20 °C until use.

### Preparation of DNA template

For the detection of oxidative damage, 100 ng of sample DNA (2 μL of stock solution) was incubated for 1 h at 37 °C in 10 μL of reaction mixture containing 1 units of FPG enzyme (New England BioLabs, UK), 10 mM Bis Tris Propane-HCl, 10 mM MgCl_2_, 1 mM DTT and 0.1 mg/mL bovine serum albumin. For detection of mtDNAcn, sample DNA was prepared in similar way, but in the absence of FPG.

### Quantitative determination of mtDNA copy number

Reactions for quantitative real-time PCR assay (qPCR) were performed with SYBR Premix EX Taq II kit (Takara, Japan) using a Rotor-Gene 6000 apparatus (Corbett Life Science, Australia). PCR was performed for 42 cycles, with a 1 min initial denaturation at 95 °C for the first cycle and all subsequent cycles consisted of denaturing at 95 °C for 4 s, annealing at 62 °C for 30 s, and extension at 72 °C for 15 s, as well as a final extension step of 15 s at 72 °C.

PCR reactions were performed in a total volume of 10 μL with 1 μL of genomic DNA, 0.5 μL of each primer, 5 μL of SYBR Premix and 3 μL of double-distilled water.

For mtDNA quantification, one set of primers targeting the mitochondrially encoded NADH dehydrogenase subunit 2 (ND2) and for nuclear DNA quantification, one set of primers targeting β-actin gene were selected. All primer were purchased from Gene Fanavaran Company (Tehran, Iran) and their exact sequences was described previously.^12^ The ratio of mtDNA to nuclear DNA in each sample was calculated as an estimate for the number of mtDNA.

### Quantitative determination of mtDNA damage

The content of guanine oxidation products was determined by qPCR-based technique. The level of oxidative damage in mitochondrial DNA is reflected by increased quantities of oxidized purine nucleotides including 8-OHdG. FPG digestion was used for the detection of oxidative DNA damage products. Levels of oxidative mtDNA damage were shown as ΔCt, which is the difference between threshold cycle (Ct) values between enzyme-treated and enzyme-nontreated samples (Ct-T and Ct-N, respectively). The higher ΔCt value corresponds to a higher levels of oxidative stress in mitochondria.

### Statistical analysis

The normally and non-normally distributed data were analyzed by independent-samples *t*-test and the Mann–Whitney *U* test, respectively. The levels of linear dependence between two variables were calculated by the Pearson correlation coefficient. This test is suitable for normal continuous variables; *p*-values of less than 0.05 were considered as threshold of statistical significance. The experimental results were shown as mean ± standard deviation. SPSS software (v11.0; SPSS, Inc. Chicago, IL, USA) was used to perform statistical operations and interpret the results of research.

## Results

Decreased mitochondrial DNA copy number in peripheral blood of patients with SSc.

**Figure 1:**
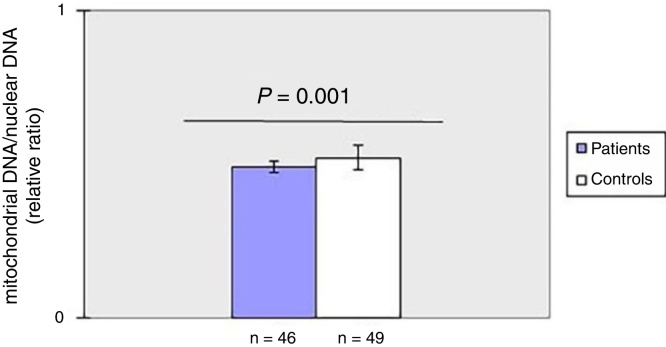
The mean mitochondrial copy number in peripheral blood of patients with systemic sclerosis and healthy subjects. Copy number of mtDNA was quantified using the delta Ct (ΔCt) of average Ct of mtDNA and nucler DNA (ΔCt = CtmtDNA + Ctβactin).

As shown in fig. 1, mtDNAcn of peripheral blood was significantly (*p* = 0.001) decreased in the patient group (0.49 ± 0.02) compared with the HC group (0.52 ± 0.04). There was no association between mtDNAcn and age, sex, disease duration. Moreover, there was no significant difference in mtDNAcn among patients with limited and diffuse subset of disease (*p* > 0.05).

### Increased oxidative DNA damage in patients with SSc

**Figure 2:**
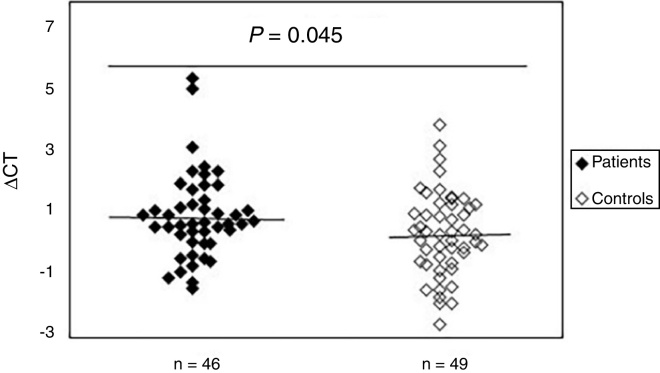
Increased mtDNA damage in patients with systemic sclerosis as compared to normal controls. The ΔCt is the difference between Ct value from DNA sample treated with FPG and Ct value from DNA sample without FPG treatment. Please note that a higher ΔCt value corresponds to a comparably higher levels of oxidative these damage.

To study the existence of oxidative damage, the presence of oxidized purine bases (mostly 8-oxodGuo) that are FPG-sensitive sites were determined in DNA of peripheral blood samples from patients with SSc and normal controls. The results showed (Fig. 2) that the mean value of oxidative DNA damage in patients (0.99 ± 1.40) was significantly increased (*p* = 0.045) compared to healthy group (0.42 ± 1.37). Moreover, no association was found between oxidative DNA damage and age, sex, and disease duration. Statistical analysis also revealed no significant differences between patients with limited and diffuse SSc in terms of oxidative DNA damage (*p* > 0.05). Interestingly, there was a negative correlation between the mtDNAcn and oxidative DNA damage (*r* = −0.392, *p* = 0.007).

## Discussion

There is general agreement that SSc is an autoimmune disease and its immnopathogenesis is similar in complexity to that of other autoimmune diseases.^13^ The exact mechanism which causes disease is unclear. Nonetheless, recent studies have indicated the significance of oxidative stress influence in disease processes. For instance, Bourji and colleagues have shown that fibrotic and nonfibrotic skins of patients with SSc have higher levels of ROS than healthy controls. They also found that SSc patients had lower vitamin C (which has powerful antioxidant activity) levels than healthy controls.^14^ Increased production of ROS by fibroblasts, endothelial and T-cells that are key cellular players in the pathogenesis of SSc suggests another evidence linking oxidative stress with the main events of the disease such as fibrosis and vascular damage.^15^

There are many different pathways for generation of ROS. Mitochondria are main sources of ROS and at the same time are the main targets of their unfavorable effects. The major bulk of mitochondrial ROS arising from the electron transport chain, as a byproduct of aerobic respiration process.^16^ ROS act as a double-edged sword because uncontrolled generation of ROS promotes oxidative stress that causes severe injury to basic kinds of biological macromolecules including DNA, protein, carbohydrates and lipid. Alternatively, ROS can participate in a variety of biological processes in normal cells including cell signaling pathways and immune responses against pathogens or foreign substances.^17^

In recent years, several studies have examined the impact of quantitative and qualitative variation of mtDNAcn on human diseases and have shown remarkable changes in mtDNAcn in various diseases.^12^, ^18^ The copy number of mitochondrial DNA can be modified by oxidative stress. Furthermore, increased production of ROS can result in oxidative damage to cellular components including DNA.^6^ Therefore, evaluation of the mtDNAcn quantity and its oxidative damage index will improve our understanding about the pathogenesis of oxidative stress-related diseases such as SSc.

The results of present study indicated that the mean value of mtDNAcn was significantly lower than those in corresponding normal. To the best of our knowledge, this report is the first of its kind to measure alterations of mtDNAcn in peripheral blood of patients with SSc. Currently, there is little information regarding the relationship between biochemical, structural, and functional changes in mitochondria and SSc.

One report by Feldmann and colleagues indicates the presence of giant mitochondria in the hepatocytes of patients with systemic scleroderma. But they did not provide any explanation concerning the mechanism(s) behind this cellular event.^19^ Some researchers have also reported the presence of Antimitochondrial Antibodies (AMAs) in the sera of patients with SSc. These antibodies are found in up to 25% of patients^20^ and have activity against components of the multi-enzyme Complex Pyruvate Dehydrogenase (PDHC)^21^ which is located in the inner mitochondrial matrix and produces reduced substrates for the electron transport chain.^22^ AMAs are considered the serum hallmark of autoimmune diseases such as Primary Biliary Cirrhosis (PBC).^23^ and the clinical motivation behind the assessment of AMAs in patients with SSc was based on the observation that a large proportion of these patients showed coexisting autoimmune condition including PBC.^24^

Several other autoantibodies, including Anticentromere (ACA), Anti-topoisomerase (ATA), and anti-RNA polymerase III (anti-RNAP) antibodies, have been described in patients with SSc that could be considered as good biomarkers for distinct clinical manifestations and prognostic subsets of SSc.^25^

There is also some evidence for a positive association between these antibodies and AMA. For instance, Wielose et al. finds that the prevalence of ACAs is significantly higher in SSc patients, who have positive AMA titers, compared with those with negative AMA titers.^26^ This study also revealed a strong association between AMAs and ACAs in patients with limited cutaneous SSc, which is consistent with previous reports.^27^

These findings indirectly suggest that mitochondria may be involved in SSc, as well as suggesting a possible role for oxidative stress. This notion is strengthened by other observations. For instance, analysis of the Bronchoalveolar Lavage Fluid (BALF) of SSc patients with lung fibrosis (SSc_Fib+_) indicated a significant upregulation of mitochondrial DNA topoisomerase 1(mtDNA TOP1),^28^ an enzyme that has a functional role in mtDNA maintenance and topology.^29^ Moreover, downregulation of Glutathione S-Transferase P (GSTP) and Superoxide Dismutase (SOD) were observed in BALF from patients with SScFib+.^28^ It must be mentioned that SOD and GSTP are two components of the anti-oxidant defence system that protect cells and tissues from oxidants especially in the lung.^30^, ^31^

Oxidative DNA base damage products also serve as markers of oxidative stress and among the four nitrogenous bases in DNA; guanine is the most easily oxidizable nucleic acid base. Therefore guanine is a primary target of oxidative modification^32^ and measurement of oxidized guanine species can provide a useful tool for assessing oxidative stress-induced DNA damage in both nuclear and mitochondrial compartments.

In the current study, we used qPCR assay to evaluate DNA base oxidation, in peripheral blood cells from SSc patients and healthy controls.

The results indicated that there is statistically a significant difference between the analyzed groups regarding oxidative mtDNA damage. Moreover, a significant negative correlation was observed between mtDNAcn and the degree of oxidative mtDNA (*r* = −0.392, *p* = 0.007).

These results are in line with previous studies that reported increased oxidative stress and DNA damage in fibroblasts obtained from patients with systemic sclerosis (scleroderma).^33^, ^34^

Although the exact molecular mechanism of DNA damage remains to be elucidated, it seems that SSc-patients derived autoantibodies can stimulate multiple intracellular signaling pathways (such as Wnt and Ras pathways) which can be influenced by ROS and can have also impact on the ROS production. In this scenario, uncontrolled changes in ROS production and signal transduction may result in accumulation of DNA damage, activation of ROS-dependent genes and high susceptibility to apoptosis. These events are thought to participate in the formation of tissue fibrosis as consequences of apoptosis and deposition of collagen.^35^

## Conclusion

In summary, the present study assessed the mtDNAcn and mtDNA damage by using a quantitative PCR-based assay. Our results show that patients with SSc reduced mtDNAcn and greater mitochondrial damage which is consistent with mitochondrial dysfunction. Therefore, in addition to traditional experiments aimed at unraveling the pathophysiology of SSc, future studies are necessary to confirm these findings.

## Financial support

This project was conducted with financial support (contract n° 24743) of Tehran University of Medical Sciences for Research.

## Authors’ contributions

Shafieh Movassaghi: Approval of the final version of the manuscript; intellectual participation in the propaedeutic and/or therapeutic conduct of the studied cases.

Sara Jafari: Approval of the final version of the manuscript; intellectual participation in the propaedeutic and/or therapeutic conduct of the studied cases.

Kowsar Falahati: Approval of the final version of the manuscript; obtaining, analysis, and interpretation of the data.

Mitra Ataei: Approval of the final version of the manuscript; obtaining, analysis, and interpretation of the data.

Mohammad Hossein Sanati: Approval of the final version of the manuscript; critical review of the manuscript.

Zohreh Jadali: Statistic analysis; approval of the final version of the manuscript; conception and planning of the study; elaboration and writing of the manuscript; obtaining, analysis, and interpretation of the data; effective participation in research orientation; intellectual participation in the propaedeutic and/or therapeutic conduct of the studied cases; critical review of the literature; critical review of the manuscript.

## Conflicts of interest

None declared.
